# First-line immune checkpoint inhibitors with chemotherapy in advanced gastric and gastroesophageal junction adenocarcinoma: a meta-analysis of phase 3 trials

**DOI:** 10.3389/fimmu.2025.1564604

**Published:** 2025-05-02

**Authors:** Yuxuan Lin, Yonghe Liao, Jinhai Shen

**Affiliations:** ^1^ Department of Pharmacy, Guangxi Hospital Division of The First Affiliated Hospital, Sun Yat-sen University, Nanning, Guangxi, China; ^2^ College of Pharmaceutical Science, Guangxi Medical University, Nanning, Guangxi, China; ^3^ State Key Laboratory of Natural Medicines, China Pharmaceutical University, Nanjing, Jiangsu, China; ^4^ Center for New Drug Safety Evaluation and Research, China Pharmaceutical University, Nanjing, Jiangsu, China

**Keywords:** immune checkpoint inhibitors, chemotherapy, gastric cancer, gastroesophageal junction adenocarcinoma, meta-analysis

## Abstract

**Background:**

The integration of immune checkpoint inhibitors (ICIs) with chemotherapy (CT) regimens has become a critical focus of clinical investigation in the management of advanced gastric and gastroesophageal junction (G/GEJ) adenocarcinoma over the past several years. Recent phase 3 trials have yielded diverse outcomes, sparking significant debate within the oncological community. In response to these disparate findings, we conducted a meta-analysis to evaluate the therapeutic efficacy and safety profile of this strategy.

**Methods:**

A literature search on PubMed and in major conference proceedings was carried out through December 15, 2024. For efficacy, summary hazard ratios (HRs) for progression-free survival (PFS) and overall survival (OS), odds ratios (ORs) for the objective response rate (ORR) were calculated; for safety, relative risks (RRs) for adverse events (AEs) were assessed.

**Results:**

Nine phase 3 clinical trials, including KEYNOTE-062, CheckMate 649, ATTRACTION-4, ORIENT-16, GEMSTONE-303, KEYNOTE-811, KEYNOTE-859, RATIONALE-305, and COMPASSION-15, which involved a total of 7,825 patients, were analyzed. The addition of ICIs to CT was associated with better PFS (HR, 0.71; 95% CI, 0.65-0.79), OS (HR, 0.79; 95% CI, 0.75-0.83), and a higher ORR (OR, 1.57; 95% CI, 1.43-1.72) compared with CT standalone treatment. However, this combination therapy increased the risk of grade 3–5 AEs (RR, 1.15; 95% CI, 1.09-1.22) and serious AEs (RR, 1.44; 95% CI, 1.21-1.70).

**Conclusion:**

For patients with advanced G/GEJ adenocarcinoma, the addition of ICIs to CT regimens as a first-line treatment offers superior efficacy compared to CT alone, though it comes with an increased risk of toxicity. In the context where multiple strategies are accessible, the pharmacological safety profile can guide practitioners in identifying the most suitable intervention for patients with a higher likelihood of deriving benefits from specific treatment strategies.

## Introduction

Gastric and gastroesophageal junction (G/GEJ) adenocarcinoma pose a major global health challenge, ranking among the most frequently diagnosed and lethal forms of cancer across the globe (1, 2). Platinum-based chemotherapy (CT) and its combination with HER-2/VEGFR-targeted therapy were the standard first-line systemic treatment options for patients with advanced G/GEJ adenocarcinoma (3–6). However, these therapeutic strategies offered only limited clinical benefits, underscoring the urgent need for innovative approaches to improve patient prognosis. Immune checkpoint inhibitors (ICIs) have demonstrated potential in treating a variety of cancers and are progressively being investigated as a therapeutic option for advanced G/GEJ adenocarcinoma (7–10).

Nine randomized controlled trials (RCTs) have evaluated first-line ICIs combinations with CT for this indication (11–22). Studies like CheckMate 649, ORIENT-16, and COMPASSION-15 reported improved overall survival (OS) and progression-free survival (PFS) with ICI-CT *vs.* CT alone, particularly in PD-L1-positive subgroups (12–14, 16, 22). Conversely, ATTRACTION-4 and KEYNOTE-811 only demonstrated PFS benefits, and KEYNOTE-062 showed no survival advantage (11, 15, 19). These discrepancies highlight unresolved questions about subgroup-specific efficacy, such as the role of PD-L1 status, HER2 positivity, or bispecific antibody use (e.g., cadonilimab in COMPASSION-15) (22). Critically, individual trials lacked statistical power to rigorously assess heterogeneous patient subgroups, leaving clinicians uncertain about optimal patient selection for ICI-CT regimens.

Against this backdrop, we conducted a meta-analysis to synthesize data from phase 3 RCTs comparing ICI-CT *vs.* CT alone in advanced G/GEJ adenocarcinoma. Our analysis aims to clarify the survival benefit of this strategy and quantify efficacy across clinically relevant subgroups—addressing the critical knowledge gap in personalized treatment decisions and guiding evidence-based guidelines for ICI integration.

## Methods

### Protocol and reporting guidelines

The research protocol was registered on the International Prospective Register of Systematic Reviews (CRD42024620712) and adhered to the Preferred Reporting Items for Systematic Reviews and Meta-analyses 2020 checklist (23).

### Information sources and search strategy

A meticulously designed systematic search strategy was implemented in PubMed to thoroughly identify all pertinent phase 3 clinical trials published up to December 15, 2024. The search formulas were constructed by combining Medical Subject Headings with free terms. The detailed queries of the search strategies are depicted in **Supplementary Table S1**. Additionally, to ensure comprehensive coverage of relevant studies, the associated conference abstracts, **Supplementary Materials**, as well as *ClinicalTrials.gov* were reviewed.

### Selection criteria

For inclusion in the meta-analysis, studies had to meet the following criteria: (i) Trials had to be phase 3 RCTs comparing the combination of ICIs and CT with CT alone. (ii) Participants were required to be patients with previously untreated advanced G/GEJ adenocarcinoma. (iii) Data on survival outcomes, including hazard ratios (HRs) with 95% confidence intervals (CIs), had to be available. Conversely, studies were excluded if: (i) They were not phase 3 RCTs. (ii) CT was not used as the control arm. (iii) ICIs were not used in the experimental arm. (iv) They were ongoing studies without published results as of the literature review date. Only studies meeting the inclusion standards were included in the meta-analysis.

### Data collection and assessment of risk of bias

The data from all included studies were extracted and summarized by one investigator and independently verified a second reviewer. The following data were collected where possible: the name of the clinical trial, the year of its publication, the size of the study sample, the treatment protocols, subgroups based on PD-L1 status, the HR along with corresponding 95% CI for PFS and OS, the odds ratio (OR) along with its associated 95% CIs for the objective response rate (ORR), and the incidence of grade 3–5 adverse events (AEs) and serious AEs. Moreover, details regarding the study design were gathered to evaluate the risk of bias in each study. The eligible studies’ bias risk was appraised thoroughly in accordance with the Cochrane bias assessment tool (24).

### Statistical analysis

The combined estimates were produced utilizing either a fixed-effects model or a random-effects model, contingent upon the level of heterogeneity observed. The *I²* statistic and Cochrane *Q* test were used to assess heterogeneity. Heterogeneity was considered significant if *I²* exceeded 50% and the *Q* test p-value was below 0.1. The choice of fixed-effects or random-effects models was guided by both statistical heterogeneity and clinical heterogeneity. Random-effects models were prioritized for outcomes with substantial clinical or methodological diversity, while fixed-effects models were used when homogeneity was strongly supported by data. To assess therapeutic efficacy, HRs with 95% CIs for PFS and OS, and ORs with 95% CIs for ORR, were calculated to obtain a pooled estimate. For the assessment of safety, relative risks (RRs) with 95% CIs for AEs were determined on a per-study basis to provide a comprehensive evaluation. Funnel plots and Egger’s tests were used to check for publication bias. Sensitivity analyses using the leave-one-out approach were conducted to validated the robustness of the pooled results. Sensitivity analyses employing the leave-one-out method were performed to assess the robustness of the pooled results. All statistical analyses were conducted using R software (version 4.2.2), with a two-tailed p-value < 0.05 considered statistically significant.

## Results

### Study selection and characteristics of included studies

The literature search yielded 42 records. Of these, nine trials satisfied the eligibility criteria and were incorporated into the analysis (11–22). The PRISMA flow diagram of identifying the eligible studies is shown in **Supplementary Figure S1**.

Among the nine included trials, one was an open-label RCT, while the remaining eight were double-blind RCTs. A total of 7,825 patients with advanced G/GEJ adenocarcinoma were included, of whom 3,922 (50.1%) received ICIs plus CT and 3,903 (49.9%) CT alone. The KEYNOTE-062 trial exclusively enrolled patients with a PD-L1 combined positive score (CPS) of ≥ 1. Separately, the KEYNOTE-811 study is designed to evaluate combination therapies in the context of HER2-positive patients. The types of ICIs used in experimental treatment regimens comprised pembrolizumab, nivolumab, sintilimab, sugemalimab, tislelizumab, and cadonilimab. The control arm regimens mainly included cisplatin plus 5-fluorouracil/capecitabine and capecitabine plus oxaliplatin. The characteristics of each trial are listed in **Table 1**.

**Table 1:** Characteristics of the included studies.

Study	Year	Design	Intention-to-treat population	PD-L1 positive subset	Regimens		Population characteristics	mPFS (Mon) HR for PFS (95% CI)	mOS (Mon) HR for OS (95% CI)
					Experimental arm	Control arm			
KEYNOTE-062 (11)	2020	Phase 3, double-blind RCT (randomization 1: 1)	507 ICT: 257 CT: 250	ICT: 99 CT: 90 (CPS ≥ 10)	Pembrolizumab plus CT	Placebo plus CT	PD-L1 CPS ≥ 1, HER2 negative, untreated, locally advanced, unresectable, or metastatic G/GEJ adenocarcinoma. 72.6% male, median age 62 years, 48% ECOG 0, 57.8% from Europe/North America/Australia, 24.2% from Asia, and 18.0% from other regions.	6.9 vs 6.4 0.84 (0.70-1.02)	12.5 vs 11.1 0.85 (0.70-1.03)
CheckMate 649 (12–14)	2021	Phase 3, open label RCT (randomization 1: 1)	1581 ICT: 789 CT: 792	ICT: 473 CT: 482 (CPS ≥ 5)	Nivolumab plus CT	CT	Untreated, unresectable, non-HER2-positive G/GEJ cancer, and esophageal adenocarcinoma. 69.5% male, 39% with age ≥ 65, 41.5% ECOG 0, 22.5% from Asia, 17% from the USA and Canada, and 61% from other regions.	7.7 vs 6.9 0.79 (0.71-0.89)	13.7 vs 11.6 0.79 (0.71-0.88)
ATTRACTION-4 (15)	2022	Phase 2-3, double-blind RCT (randomization 1: 1)	724 ICT: 362 CT: 362	ICT: 58 CT: 56 (TPS ≥ 1%)	Nivolumab plus CT	Placebo plus CT	HER2 negative, untreated, unresectable advanced or recurrent G/GEJ adenocarcinoma. 72.5% male, 50.8% with age ≥ 65, 54% ECOG 0, 55% from Japan, 41% from South Korea, and 4% from Taiwan.	10.45 vs 8.34 0.68 (0.51-0.90)	17.45 vs 17.15 0.90 (0.75-1.08)
ORIENT-16 (16)	2023	Phase 3, double-blind RCT (randomization 1: 1)	650 ICT: 327 CT: 323	ICT: 197 CT: 200 (CPS ≥ 5)	Sintilimab plus CT	Placebo plus CT	Unresectable, no known HER2 positive status, locally advanced or metastatic G/GEJ adenocarcinoma. 74.3% male, 36.1% with age ≥ 65 years, 27.7% ECOG 0, all patients were from China.	7.1 vs 5.7 0.64 (0.52-0.77)	15.2 vs 12.3 0.77 (0.63-0.94)
GEMSTONE-303 (17, 18)	2023 2025	Phase 3, double-blind RCT (randomization 1: 1)	479 ICT: 241 CT: 238	ICT: 130 CT: 128 (CPS ≥ 10)	Sugemalimab plus CT	Placebo plus CT	Untreated, no known HER2-positive status, PD-L1 expression ≥ 5%, unresectable advanced or metastatic G/GEJ adenocarcinoma. 73.1% male, 43.2% with age ≥ 65 years, 24.6% ECOG 0, all patients were from China.	7.62 vs 6.08 0.66 (0.54-0.81)	15.64 vs 12.65 0.75 (0.61-0.92)
KEYNOTE-811 (19)	2023	Phase 3, double-blind RCT (randomization 1: 1)	698 ICT: 350 CT: 348	ICT: 298 CT: 296 (CPS ≥ 1)	Pembrolizumab plus trastuzumab and CT	Placebo plus trastuzumab and CT	Untreated, locally advanced or metastatic HER2 positive G/GEJ adenocarcinoma. 81% male, 41% with age ≥ 65 years, 42% ECOG 0, 32% from Australia, Europe, Israel, and North America; 34% from Asia; and 34% from the rest of the world.	10.0 vs 8.1 0.73 (0.61-0.87)	20.0 vs 16.8 0.84 (0.70-1.01)
KEYNOTE-859 (20)	2023	Phase 3, double-blind RCT (randomization 1: 1)	1579 ICT: 790 CT: 789	ICT: 618 CT: 617 (CPS ≥ 1)	Pembrolizumab plus CT	Placebo plus CT	Untreated, locally advanced or metastatic HER2 negative G/GEJ adenocarcinoma. 67. 8% male, 39% with age ≥ 65 years, 37% ECOG 0, 33% from Asia, 25% from Western Europe, Israel, North America, and Australia, and 41% from the rest of the world.	6.9 vs 5.6 0.76 (0.67-0.85)	12.9 vs 11.5 0.78 (0.70-0.87)
RATIONALE-305 (21)	2024	Phase 3, double-blind RCT (randomization 1: 1)	997 ICT: 501 CT: 496	ICT: 274 CT: 272 (TAP ≥ 5%)	Tislelizumab plus CT	Placebo plus CT	HER2 negative locally advanced unresectable or metastatic G/GEJ adenocarcinoma. 69.5% male, 35% with age ≥ 65 years, 32.4% ECOG 0, 75% from Asia, and 25% from Europe and North America.	6.9 vs 6.2 0.78 (0.67-0.90)	15.0 vs 12.9 0.80 (0.70-0.92)
COMPASSION-15 (22)	2025	Phase 3, double-blind RCT (randomization 1: 1)	610 ICT: 305 CT: 305	ICT: 122 CT: 123 (CPS ≥ 5)	Cadonilimab plus CT	Placebo plus CT	Untreated, HER2 negative unresectable locally advanced or metastatic G/GEJ adenocarcinoma. 77.7% male, 45.2% with age ≥ 65 years, 23.3% ECOG 0, all patients were from China.	7.0 vs 5.3 0.53 (0.44-0.65)	15.0 vs 10.8 0.62 (0.50-0.78)

ICT, immunochemotherapy; CT, chemotherapy; RCT, randomized clinical trial; CPS, combined positive score; TPS, tumor cell proportion score; TAP, tumor area positivity; G/GEJ, gastric and gastroesophageal junction; mPFS, median progression-free survival; mOS, median overall survival; HR, hazard ratio; CI, confidence interval.

### Efficacy in intention-to-treat population

**Figure 1:**
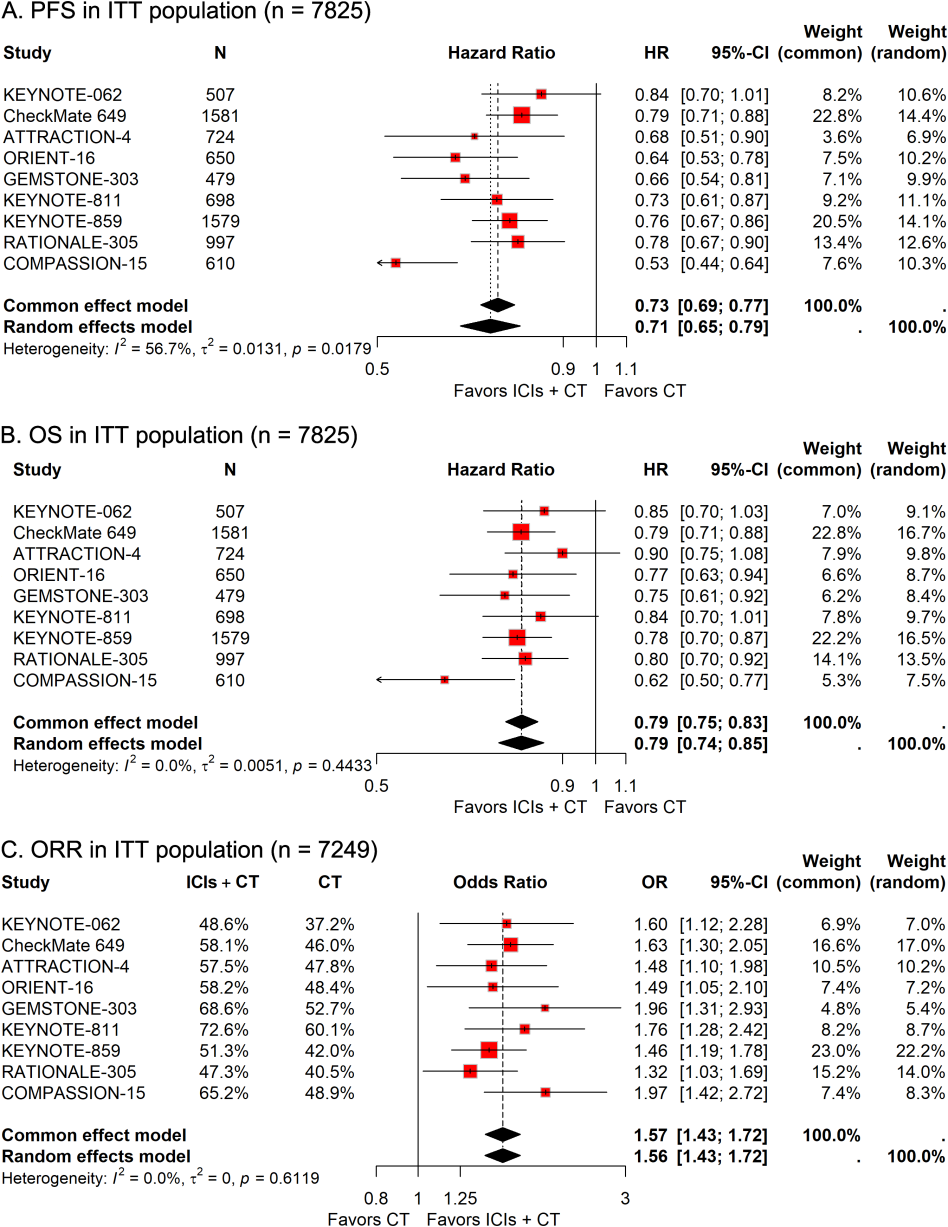
Forest plots depicting pooled efficacy outcomes in the intention-to-treat (ITT) population: **(A)** progression-free survival (PFS), **(B)** overall survival (OS), and **(C)** objective response rate (ORR) for immune checkpoint inhibitors plus chemotherapy (ICIs + CT) *versus* chemotherapy (CT) alone.

Data for PFS and OS were available from all nine trials involving 7,825 patients. The combined HR for PFS suggested a significant improvement with ICI-CT compared to CT alone (HR, 0.71; 95% CI, 0.65-0.79; **Figure 1A**), corresponding to a 29% relative reduction in disease progression risk. For OS, the combined HR indicated a 21% relative reduction in mortality risk with ICI-CT (HR, 0.79; 95% CI, 0.75-0.83; **Figure 1B**). ORR data from 7,249 patients showed a 57% increase in response rate with ICI-CT (OR, 1.57; 95% CI, 1.43-1.72; **Figure 1C**). These findings demonstrate consistent efficacy of ICI-CT across survival and response endpoints.

### Safety in ITT population

**Figure 2:**
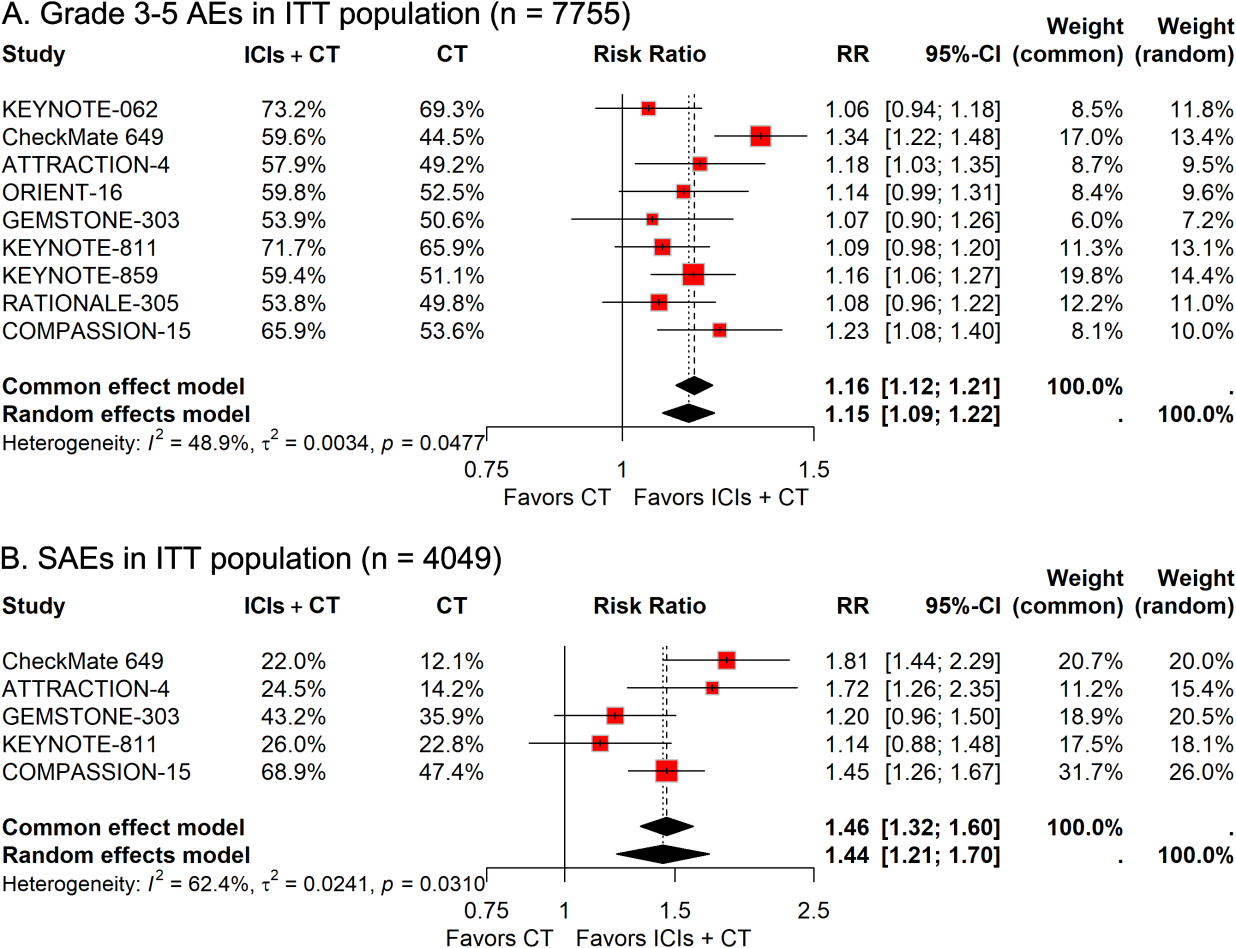
Forest plots illustrating safety outcomes in the intention-to-treat (ITT) population: **(A)** grade 3–5 adverse events (AEs) and **(B)** serious AEs, reported as relative risk (RR) for immune checkpoint inhibitors plus chemotherapy (ICIs + CT) *versus* chemotherapy (CT) alone.

In the group of 3,898 patients receiving ICIs combined with CT, 2,369 individuals (60.8%) suffered from grade 3–5 AEs, compared to 2,013 of the 3,857 patients (52.2%) treated only with CT. The pooled RR revealed that the addition of ICIs to CT significantly elevated the risk of grade 3–5 AEs (RR, 1.15; 95% CI, 1.09-1.22; **Figure 2A**). Safety data regarding serious AEs of ICIs plus CT *versus* CT alone were available in five trials. The prevalence of serious AEs was 32.6% (665/2,037) in the ICIs plus CT group and 22.5% (452/2,012) in the CT-alone group. The pooled analysis results indicated that patients receiving the combination treatment had a significantly increased risk of experiencing serious AEs (RR, 1.44; 95%CI, 1.21-1.70; **Figure 2B**).

### Subgroup analysis

Due to the fact that individual studies lacked sufficient power to analyze diverse clinically related subgroups, to better understand the efficacy of ICIs combined with CT in specific subsets and guide individualized precision treatment, we performed a series of subgroup analyses according to patient demographics, disease pathology, and therapeutic protocols

#### Subgroup analysis stratified by patient demographics

**Figure 3:**
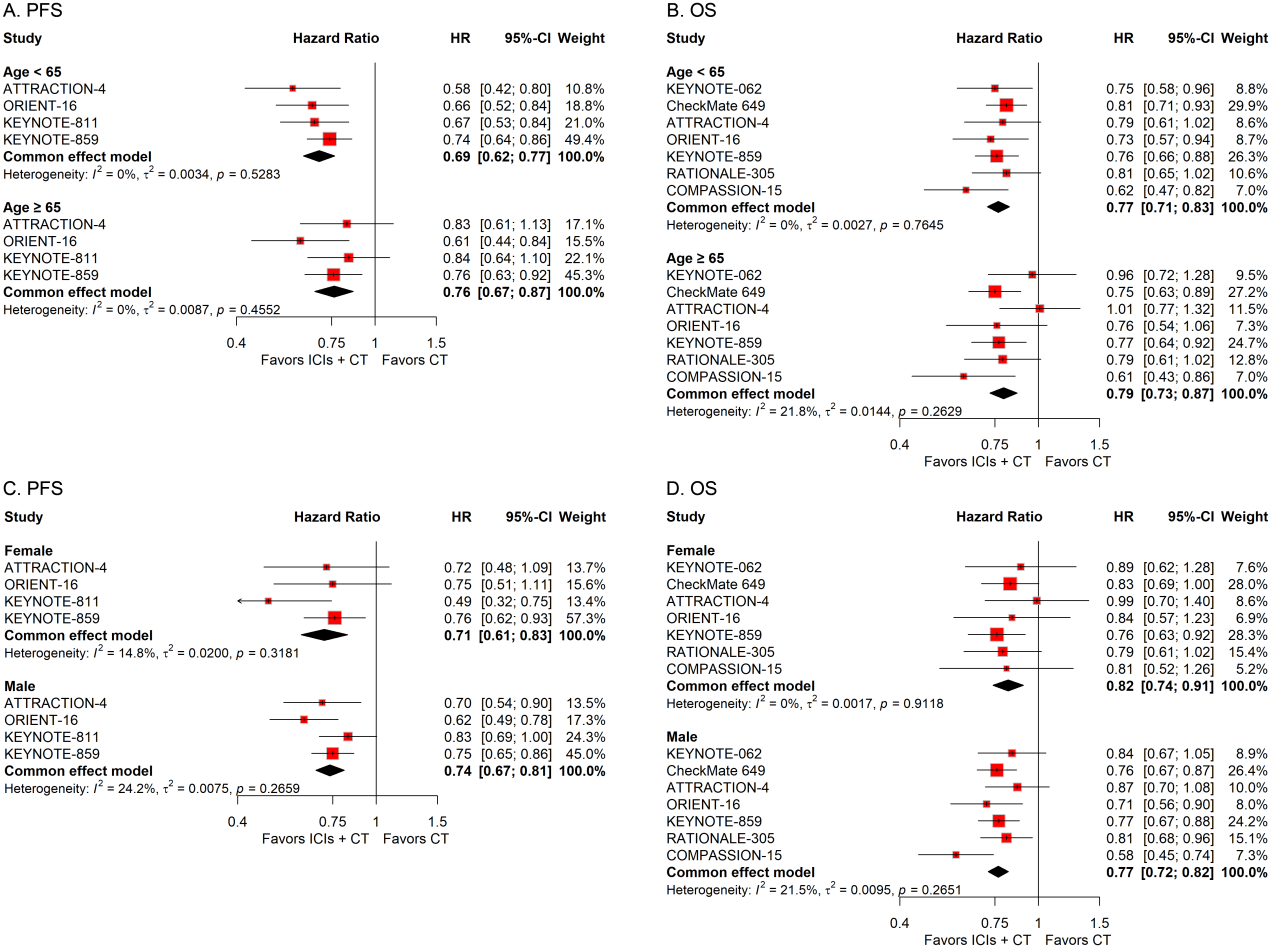
Forest plots displaying the results of progression-free survival (PFS) and overall survival (OS) stratified by **(A, B)** age and **(C, D)** sex in patients receiving immune checkpoint inhibitors plus chemotherapy (ICIs + CT) *versus* chemotherapy (CT) alone.

**Figure 4:**
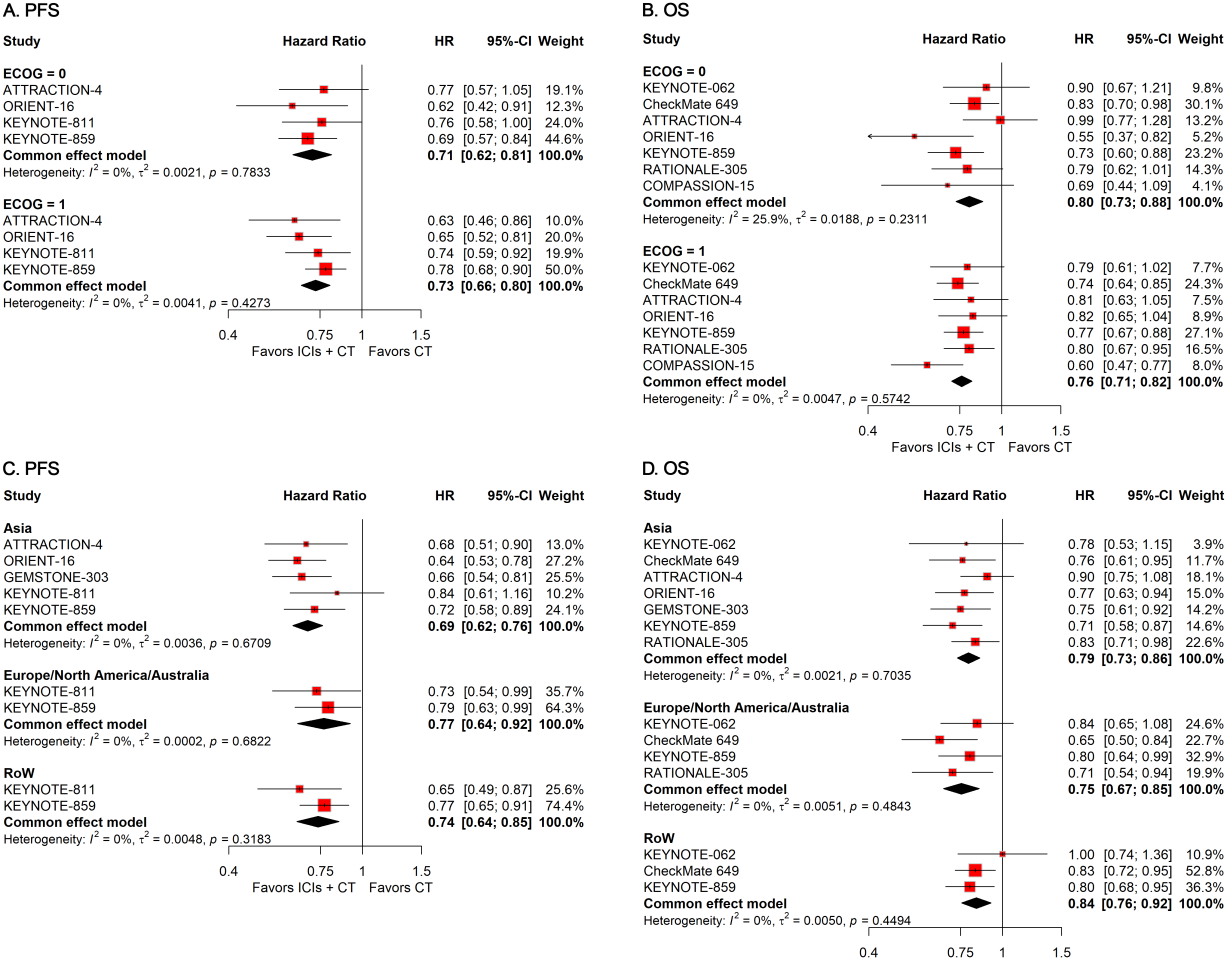
Forest plots displaying the results of progression-free survival (PFS) and overall survival (OS) stratified by **(A, B)** Eastern Cooperative Oncology Group (ECOG) performance status and **(C, D)** residential area.

To investigate how differences in demographic features affect the therapeutic efficacy of ICIs plus CT in G/GEJ adenocarcinoma patients, we carried out various subgroup evaluations taking into account relevant factors such as age, sex, Eastern Cooperative Oncology Group (ECOG) Performance Status, and residential area. The pooled HRs revealed that the addition of ICIs to CT significantly prolonged survival regardless of age, sex, ECOG status, and residential area (**Figures 3**, **4**).

#### Subgroup analysis stratified by disease pathology

**Figure 5:**
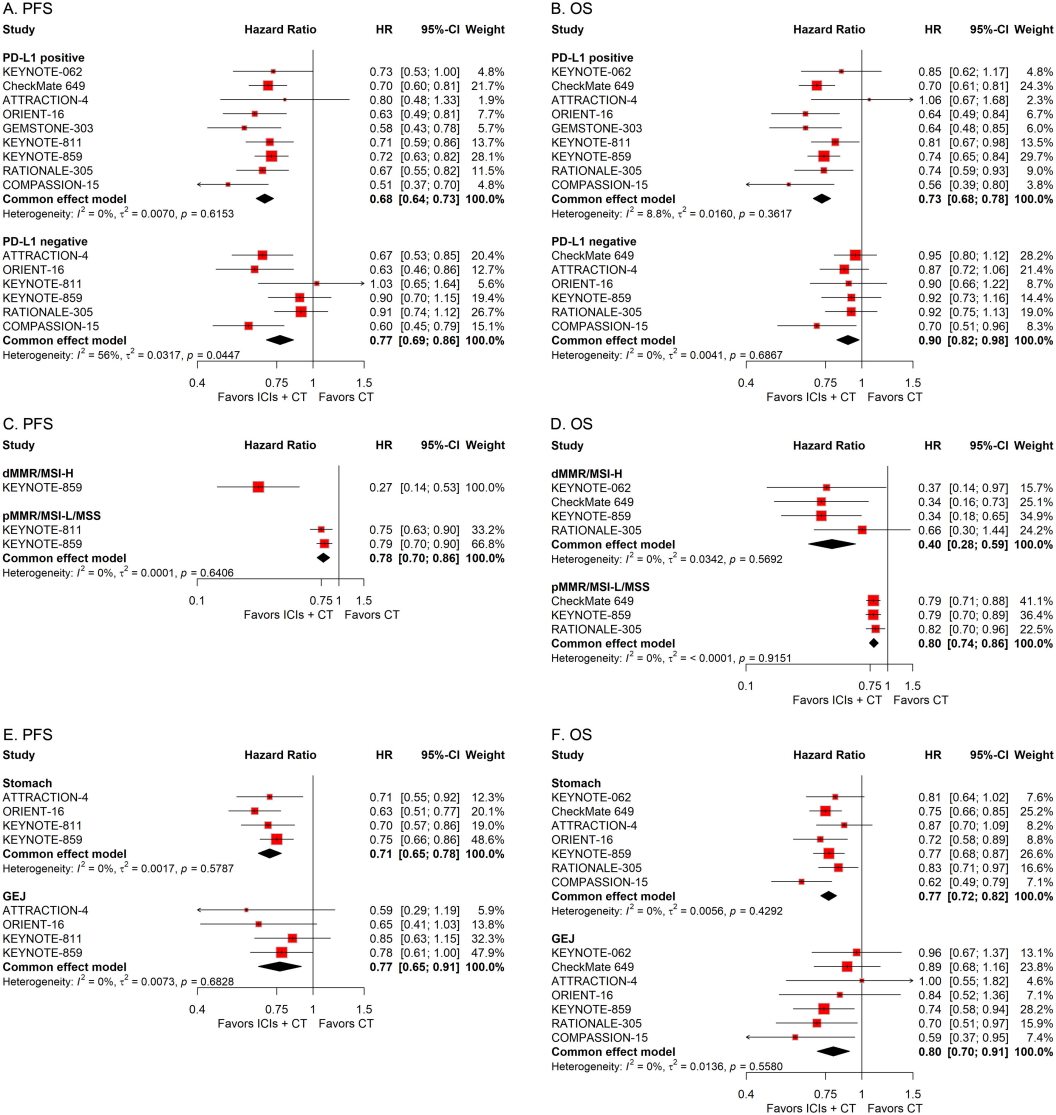
Forest plots displaying the results of progression-free survival (PFS) and overall survival (OS) stratified by PD-L1 status **(A, B)**, microsatellite status **(C, D)**, and tumor site **(E, F)**.

**Figure 6:**
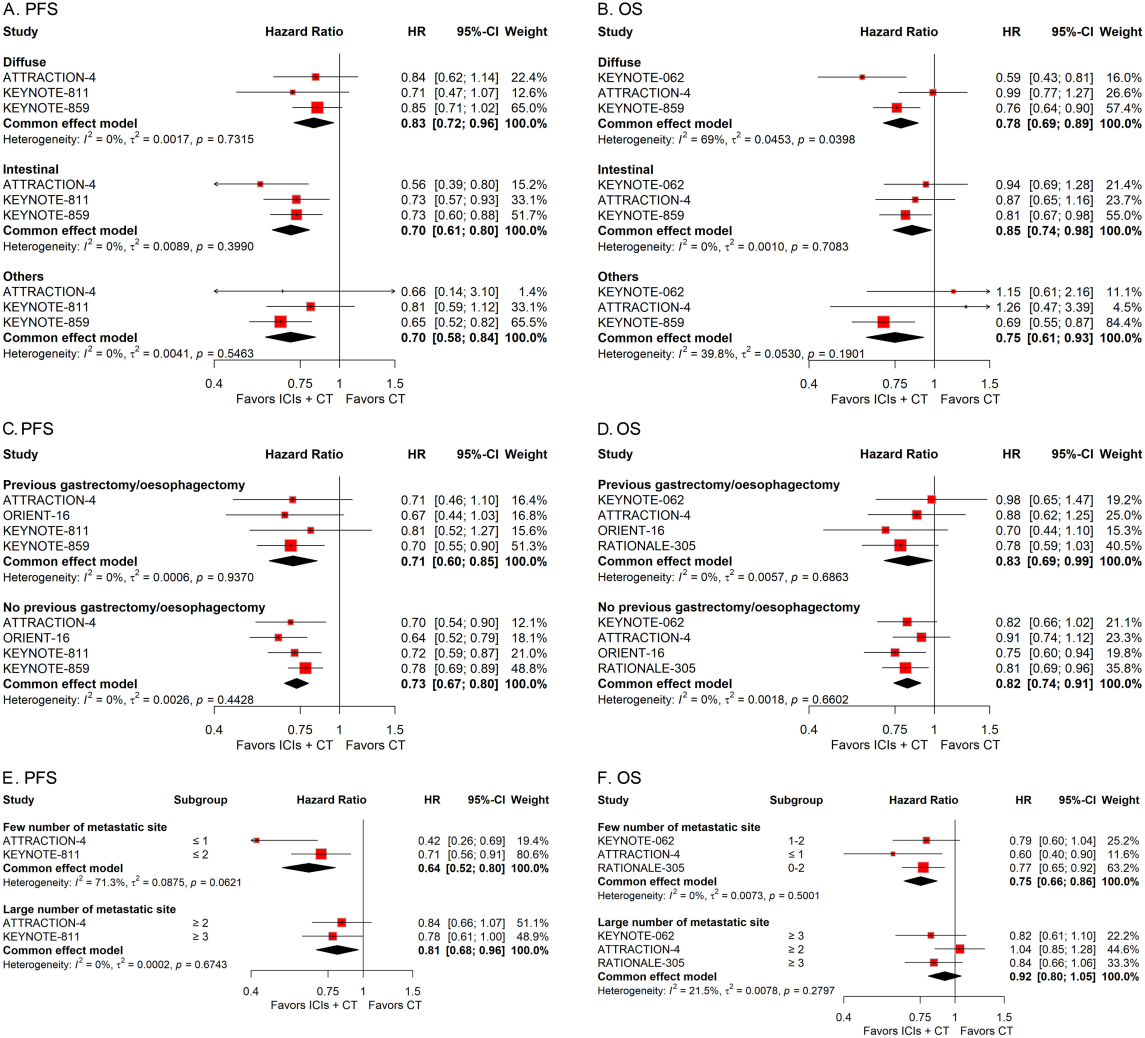
Forest plots displaying the results of progression-free survival (PFS) and overall survival (OS) stratified by subtype **(A, B)**, history of previous gastrectomy or esophagectomy **(C, D)**, and number of metastasis sites **(E, F)**.

To explore the influence of diverse pathological attributes on the therapeutic outcome of ICIs in conjunction with CT for G/GEJ adenocarcinoma, we performed a series of subgroup assessments based on various factors such as PD-L1 status, microsatellite status, tumor site, subtype, history of previous gastrectomy or esophagectomy, and number of metastasis sites. The combined HRs indicated a substantial enhancement in survival with the integration of ICIs and CT regardless of PD-L1 status, microsatellite status, tumor site, subtype, and history of previous gastrectomy or esophagectomy (**Figures 5**, **6**). However, while the combination of ICIs and CT significantly improved PFS regardless of the number of metastasis sites, it only improved OS in patients with a few metastasis sites rather than a large number of them (**Figures 6E, F**).

#### Subgroup analysis stratified by treatment protocols

**Figure 7:**
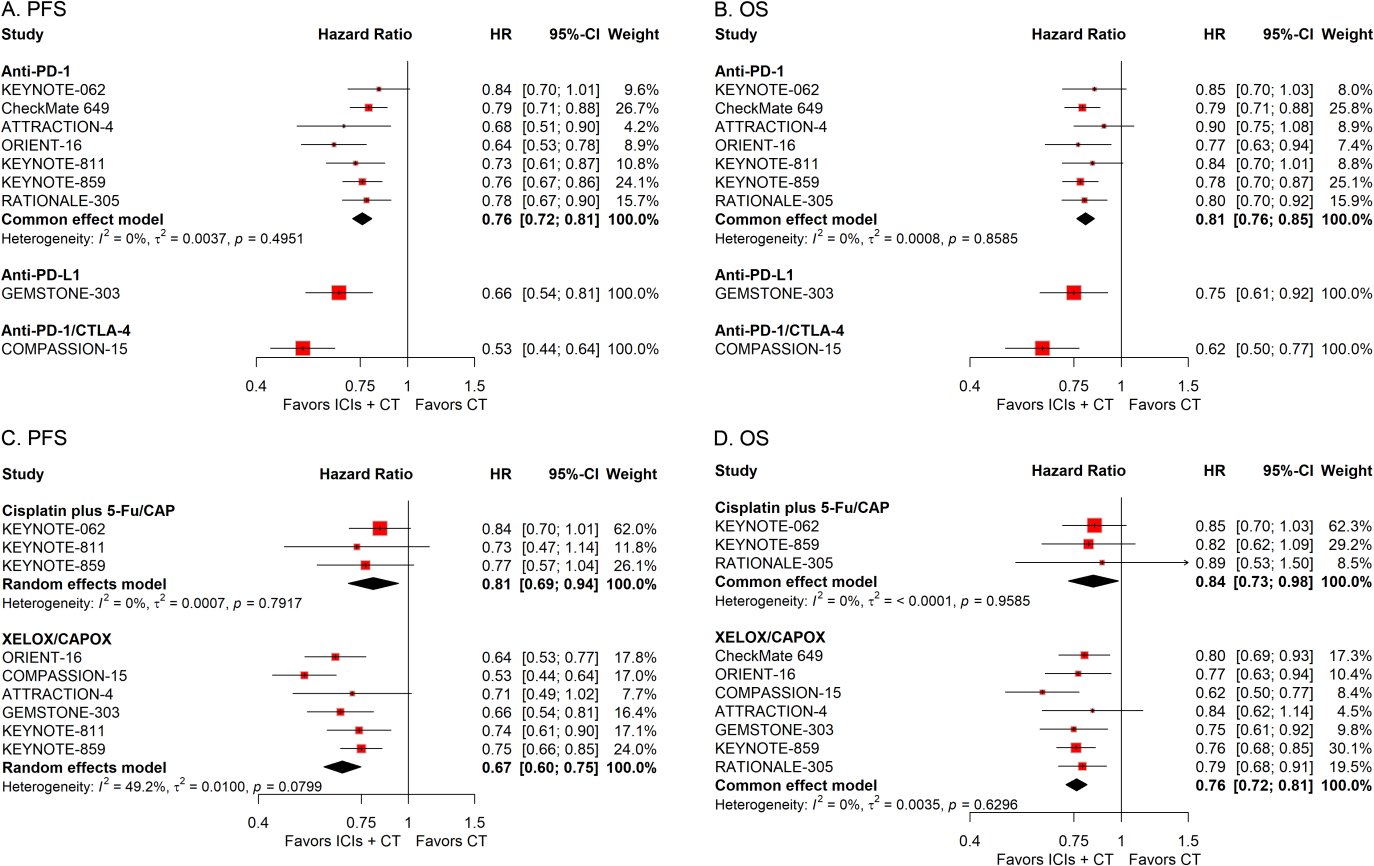
Forest plots displaying the results of progression-free survival (PFS) and overall survival (OS) stratified by the type of ICIs **(A, B)** and the regimen of CT **(C, D)**.

To examine the effects of different treatment protocols on the efficacy of ICIs combined with CT for G/GEJ adenocarcinoma, we undertook two separate subgroup studies, focusing on variables including the ICIs classification and the CT treatment scheme. The pooled HRs revealed that the addition of ICIs to CT notably extended survival regardless of the type of ICIs and regimen of CT used (**Figure 7**).

### Risk of bias and sensitivity analysis

Details of the bias risk assessment for each study were presented in **Supplementary Figures S2**, **S3**. No substantial publication bias was detected (**Supplementary Figure S4**). Sensitivity analyses verified the stability of the combined outcomes. (**Supplementary Figure S5**).

## Discussion

The addition of ICIs to CT regimens for advanced cancers has emerged as a pivotal area of investigation within oncology (10, 25), with its role in G/GEJ adenocarcinoma garnering significant attention. Several phase 3 clinical trials have previously explored the efficacy of ICIs in combination with CT for advanced G/GEJ adenocarcinoma. While individual studies presented variable results, particularly in terms of OS, the overall trend points towards a benefit with the addition of ICIs. This meta-analysis aimed to integrate data from multiple phase 3 clinical trials to deeply explore the efficacy and safety of this combined treatment strategy, providing a strong basis for clinical decision-making.

The results of our meta-analysis unequivocally demonstrate the significant enhancement in PFS, OS, and ORR when ICIs are added to CT compared to CT alone. These results not only reinforce the therapeutic potential of ICIs in advanced G/GEJ adenocarcinoma but also suggest that ICIs combined with CT have the potential to emerge as a hopeful new benchmark in the treatment of this difficult-to-treat and frequently resistant cancer. However, while the efficacy benefits are clear, the significant increase in toxicity must be carefully considered. The higher risk of grade 3–5 AEs and serious AEs associated with the ICIs plus CT regimen calls for heightened vigilance in monitoring patients. The toxicity profile of this combination highlights the need for individualized management strategies. Strategies to manage these AEs, such as early detection and prompt intervention, will be essential in maximizing the therapeutic benefits without compromising patient safety. In clinical practice, patients’ individual situations should be fully considered, the benefits and risks of treatment should be balanced, and reasonable treatment plans should be formulated. At the same time, multidisciplinary collaboration should be strengthened to provide comprehensive treatment and management for patients. In addition, continuous attention should be paid to research progress, and new research achievements should be timely applied to clinical practice to continuously optimize treatment strategies and improve patients’ treatment outcomes and quality of life.

The subgroup analyses provide valuable insights into the potential variability in treatment response across different patient subgroups. The consistent improvement in PFS and OS across various demographic and pathological characteristics, including age, sex, ECOG performance status, PD-L1 status, microsatellite status, tumor site, subtype, and history of previous gastrectomy or esophagectomy, suggests that ICI plus CT may benefit a broad spectrum of patients with advanced G/GEJ adenocarcinoma. However, the observed difference in OS benefit based on the number of metastasis sites highlights the need for personalized treatment approaches. Patients with fewer metastasis sites may experience greater OS benefits from ICI plus CT, suggesting a potential role for this combination in earlier-stage disease or in patients with a limited burden of metastatic disease.

While this meta-analysis provides valuable insights into the efficacy and safety of ICIs plus CT in advanced G/GEJ adenocarcinoma, there are several limitations that should be acknowledged. First, the included trials had certain heterogeneity in patient populations, treatment regimens, PD-L1 assessment methods, which might affect the accuracy and universality of the results. Although random-effects model was used to handle heterogeneity in the analysis, the existence of heterogeneity remains a non-negligible issue. The included trials utilized varying PD-L1 evaluation metrics, such as CPS, tumor proportion score (TPS), and tumor area positivity (TAP), with different cutoff values (e.g., CPS ≥ 1 *vs.* CPS ≥ 10, TPS ≥ 1%) (11, 15, 22). For example, KEYNOTE-062 enrolled patients with CPS ≥ 1, while GEMSTONE-303 required CPS ≥ 10, reflecting divergent criteria for defining PD-L1 positivity. This inconsistency may have introduced bias in subgroup analyses, as PD-L1 expression thresholds are known to correlate with ICIs response rates (12, 19). Notably, our subgroup analysis showed consistent PFS/OS benefits across all PD-L1 strata, but the lack of uniform assessment methods limits the precision of biomarker-driven conclusions. Future trials should standardize PD-L1 evaluation to facilitate comparable subgroup analyses. CT backbones and ICI types varied significantly across trials. CT regimens included cisplatin plus 5-fluorouracil, capecitabine plus oxaliplatin, and other combinations, while ICIs encompassed PD-1 inhibitors (pembrolizumab, nivolumab, sintilimab, tislelizumab), a PD-L1 inhibitor (sugemalimab), and a dual PD-1/CTLA-4 bispecific antibody (cadonilimab in COMPASSION-15) (12, 22). These differences may affect efficacy and toxicity profiles: for instance, dual checkpoint inhibition in COMPASSION-15 showed particularly pronounced OS benefits (HR = 0.62) but was not directly comparable to single-agent PD-1 inhibitors (22). Additionally, HER2-targeted combinations (e.g., trastuzumab in KEYNOTE-811) introduced further complexity, as HER2 positivity itself is a prognostic factor (19). Despite this diversity, our subgroup analysis indicated consistent benefits across ICI classes and CT regimens, suggesting a robust overall effect of chemo-immunotherapy combinations. However, the safety data (e.g., grade 3–5 AEs) may be influenced by regimen intensity, highlighting the need for tailored toxicity management based on specific ICI-CT combinations. The trials enrolled patients from diverse regions, including Asia, Europe, and North America, where gastric cancer epidemiology (e.g., Helicobacter pylori infection rates, tumor subtypes) and treatment practices differ (2, 15). For example, ATTRACTION-4 and ORIENT-16 recruited primarily Asian populations, while CheckMate 649 included a more global cohort (12, 15). Ethnic differences in immune response pathways (e.g., HLA genotypes) and baseline comorbidities may contribute to treatment variability, though our subgroup analysis by residential area showed uniform benefits across regions (**Figures 4C, D**). Nonetheless, underreporting of geographic-specific data in some trials limits the ability to fully dissect these effects, emphasizing the need for stratified analyses in future multinational studies. These sources of heterogeneity highlight the real-world complexity of treating advanced G/GEJ adenocarcinoma, where patient selection and regimen choice are influenced by multiple factors. While our random-effects model provided conservative estimates applicable to broad populations, clinicians should interpret subgroup results with awareness of biomarker assay variability and treatment context. Moving forward, standardizing PD-L1 assessment (e.g., adopting CPS as a unified metric), reporting detailed geographic/ethnic stratification, and investigating interactions between ICI types and CT backbones will be crucial for optimizing precision oncology in this setting. Additionally, while the analysis was based on phase 3 clinical trials, the duration of follow-up in many of these studies was relatively short. Longitudinal assessments of patient survival, quality of life, and treatment tolerability will be crucial in informing clinical decision-making and refining treatment algorithms for these types of cancers. Finally, although specific biases (e.g., open-label design) exist in individual trials, the predominance of low-risk studies for key bias domains (**Supplementary Figures S2**, **S3**), absence of publication bias, and stable results in sensitivity analyses (**Supplementary Figures S4**, **S5**) reinforce the robustness of our conclusions.

While our meta-analysis demonstrates significant survival benefits of ICI-CT combinations in controlled clinical trials, their translation into real-world practice requires careful consideration. Recent real-world studies report consistent efficacy but highlight key differences. A multicenter retrospective cohort (n = 573) showed even stronger survival benefits (PFS: HR = 0.45, OS: HR = 0.40), likely due to less post-progression crossover and broader patient inclusion, including elderly (≥ 75 years) and PD-L1-unknown patients (60% of the cohort) (26). Notably, patients with low PD-L1 CPS (1-4) achieved significant OS prolongation (HR = 0.24), filling an evidence gap from RCTs that focus on CPS ≥ 5. However, real-world challenges include higher treatment discontinuation in elderly patients due to toxicity and limited access to specialty care for managing immune-related adverse events. Economic barriers further impact equity, particularly in regions with low PD-L1 testing rates. These insights complement our meta-analysis, advising clinicians to balance efficacy with patient-specific factors—such as comorbidities, testing availability, and resource constraints—when implementing ICI-CT, especially for patients with unclear biomarker status or complex real-world profiles. This integrated evidence supports a pragmatic approach to optimize treatment selection while advocating for standardized toxicity protocols and policy interventions to enhance accessibility.

The promising results of this meta-analysis open several avenues for future research. First, while clinical trials have highlighted the importance of biomarkers such as PD-L1 expression, further refinement in biomarker-guided treatment will be essential for optimizing patient selection and improving therapeutic outcomes. The KEYNOTE-062 trial, for example, demonstrated that pembrolizumab monotherapy provided a significant benefit for patients with PD-L1-positive tumors, yet the combination of pembrolizumab and CT failed to significantly extend PFS or OS over solo CT treatment. This finding suggests the importance of PD-L1 expression in guiding treatment decisions, especially as we continue to explore the efficacy of ICIs in different subsets of G/GEJ adenocarcinoma. Thus, while PD-L1 remains an important biomarker for identifying potential responders to immunotherapy, the role of other biomarkers (e.g., microsatellite instability, tumor mutational burden) and clinical features will need to be further explored. In addition, the exploration of dual immune checkpoint inhibition (e.g., combining PD-1/PD-L1 inhibitors with CTLA-4 inhibitors) in combination with CT is an area of active investigation. The COMPASSION-15 trial, which evaluated the combination of cadonilimab, an anti-PD-1 and anti-CTLA-4 bispecific antibody, with CT, demonstrated promising results in improving PFS both and OS, highlighting the potential for more aggressive immunotherapy combinations in the future. However, the balance between efficacy and toxicity with dual immune checkpoint inhibition requires further investigation. Moreover, future studies should aim to explore precision medicine approaches that integrate genetic, molecular, and immune profiles of patients to better predict responses to ICIs. While current research has identified some potential biomarkers, such as PD-L1 expression and microsatellite instability, more comprehensive profiling of the tumor microenvironment and immune landscape may allow for more precise patient selection. Finally, given the promising results of ICIs in HER2-positive G/GEJ adenocarcinoma in the KEYNOTE-811 trial, further exploration of immunotherapy in combination with targeted therapies, such as HER2 inhibitors (trastuzumab), could represent a future frontier for treating this specific patient subset.

## Conclusion

The addition of ICIs to CT demonstrates robust improvements in PFS, OS, and ORR, confirming the efficacy of this combination approach in the treatment of advanced G/GEJ adenocarcinoma. However, the heightened risk of toxicity underscores the importance of meticulous patient selection and tailored management strategies. Subgroup analysis indicates that the combination of ICIs and CT may confer benefits across various patient subgroups. Given the availability of several strategies in this setting, the safety profile of drugs and their costs may assist clinicians in choosing the most suitable treatment for patients with G/GEJ adenocarcinoma who are prone to gain advantages from the treatment modality.

## Data Availability

The original contributions presented in the study are included in the article/**Supplementary Material**. Further inquiries can be directed to the corresponding author.
