# Supplementary material for: First-line immune checkpoint inhibitors with chemotherapy in advanced gastric and gastroesophageal junction adenocarcinoma: a meta-analysis of phase 3 trials
Source: Front Immunol. 2025 May 2;16:1564604. doi: 10.3389/fimmu.2025.1564604 (PMC12081356; doi:10.3389/fimmu.2025.1564604)
Supplement: Supplementary file 1 [file DataSheet1.docx]

**Supplementary Material**

**Addition of immune checkpoint inhibitors to first-line chemotherapy in advanced gastric or gastroesophageal junction adenocarcinoma: A meta-analysis of phase 3 clinical trials**

**Table S1.** Details of searching strategy.

**Figure S1.** PRISMA flow diagram of study selection.

**Figure S2.** Risk of bias graph.

**Figure S3.** Risk of bias summary.

**Figure S4.** Funnel plots and Egger’s tests for progression-free survival, overall survival, and objective response rate.

**Figure S5.** Sensitivity analyses for included studies on progression-free survival, overall survival, and objective response rate examined by leaving-one-out approach.

**Table S1.** Details of searching strategy.

| **Database** | **Search strategy** |
| --- | --- |
| ***PubMed*** | (("Stomach Neoplasms"[MeSH Terms] OR "gastric cancer"[All Fields] OR "gastro-oesophageal junction adenocarcinoma"[All Fields] OR "gastroesophageal junction adenocarcinoma"[All Fields] OR "gastric or gastro-oesophageal junction adenocarcinoma"[All Fields]) AND ("Immune Checkpoint Inhibitors"[MeSH Terms] OR ("immunotherapy"[MeSH Terms] OR "immunotherapy"[All Fields] OR "immunotherapies"[All Fields] OR "immunotherapy s"[All Fields]) OR "PD-1"[All Fields] OR "PD-L1"[All Fields] OR ("ctla 4 antigen"[MeSH Terms] OR ("ctla 4"[All Fields] AND "antigen"[All Fields]) OR "ctla 4 antigen"[All Fields] OR "ctla 4"[All Fields])) AND ("chemotherapy s"[All Fields] OR "drug therapy"[MeSH Terms] OR ("drug"[All Fields] AND "therapy"[All Fields]) OR "drug therapy"[All Fields] OR "chemotherapies"[All Fields] OR "drug therapy"[MeSH Subheading] OR "chemotherapy"[All Fields])) AND (clinicaltrialphaseiii[Filter]) |
| ***ASCO, ESMO, and AACR*** | ((anti-CTLA-4) OR (ipilimumab) OR (anti-PD-1) OR (anti-PDL1) OR (toripalimab) OR (pembrolizumab) OR (nivolumab) OR (atezolizumab) OR (avelumab) OR (durvalumab) OR (tremelimumab) OR (sintilimab) OR (camrelizumab) OR ("immune checkpoint inhibitor*")) AND (chemotherapy) AND ("gastric cancer" OR "gastro-oesophageal junction adenocarcinoma" OR "gastric or gastro-oesophageal junction adenocarcinoma") |

ASCO, American Society of Clinical Oncology; ESMO, European Society for Medical Oncology; AACR, American Association for Cancer Research.


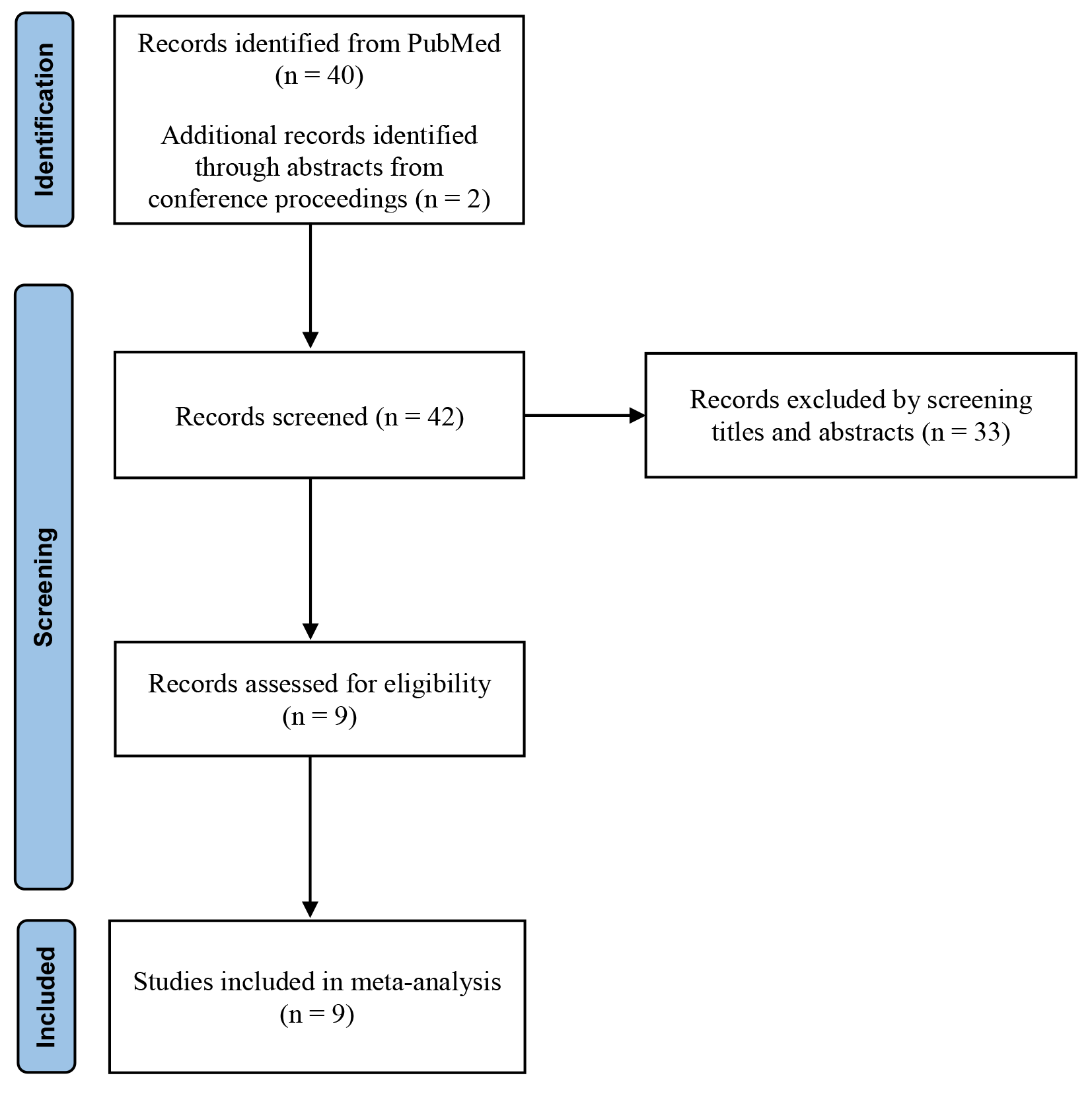


**Figure. S1.** PRISMA flow diagram of study selection.

**
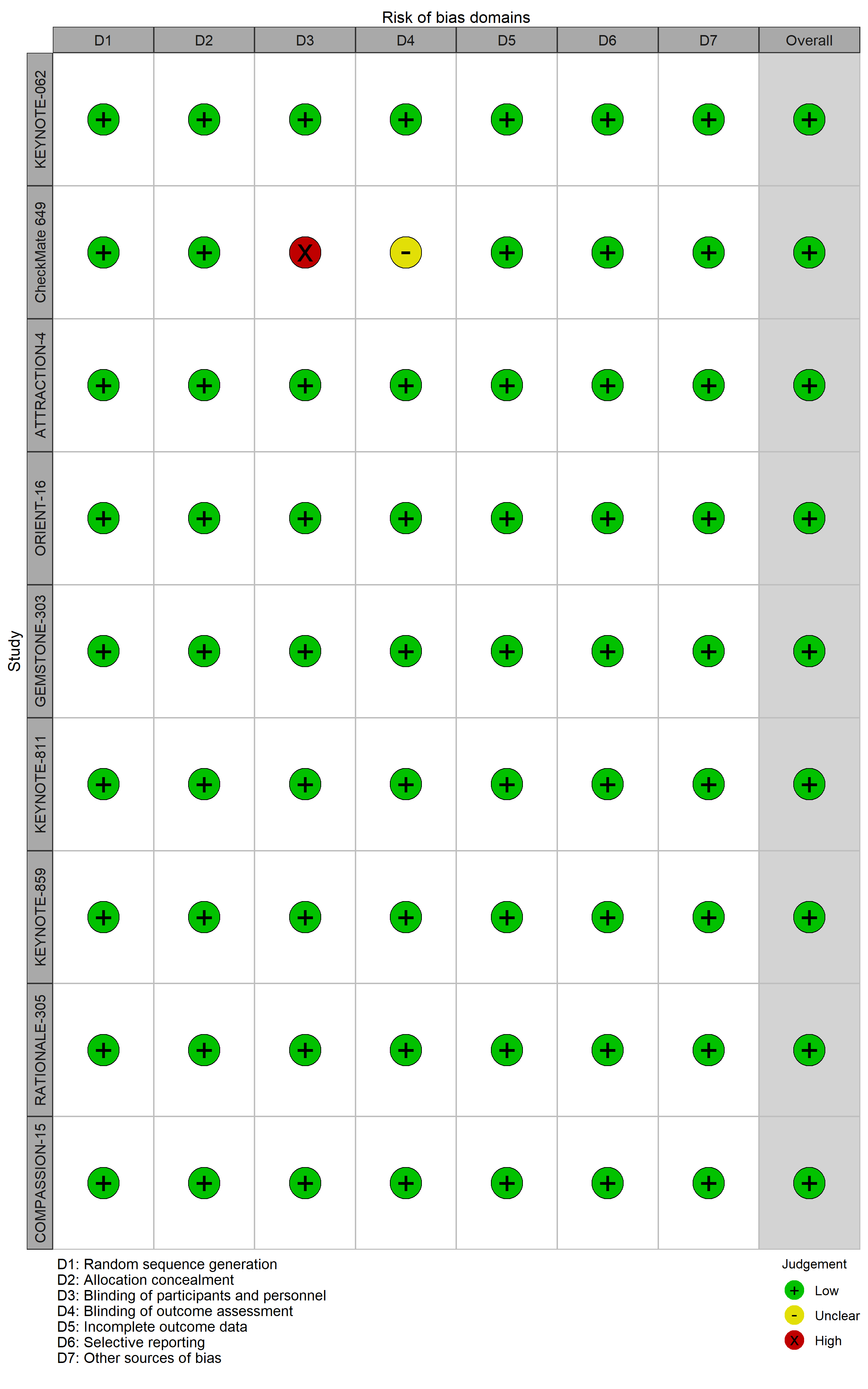
**

**Figure. S2.** Risk of bias graph.

**
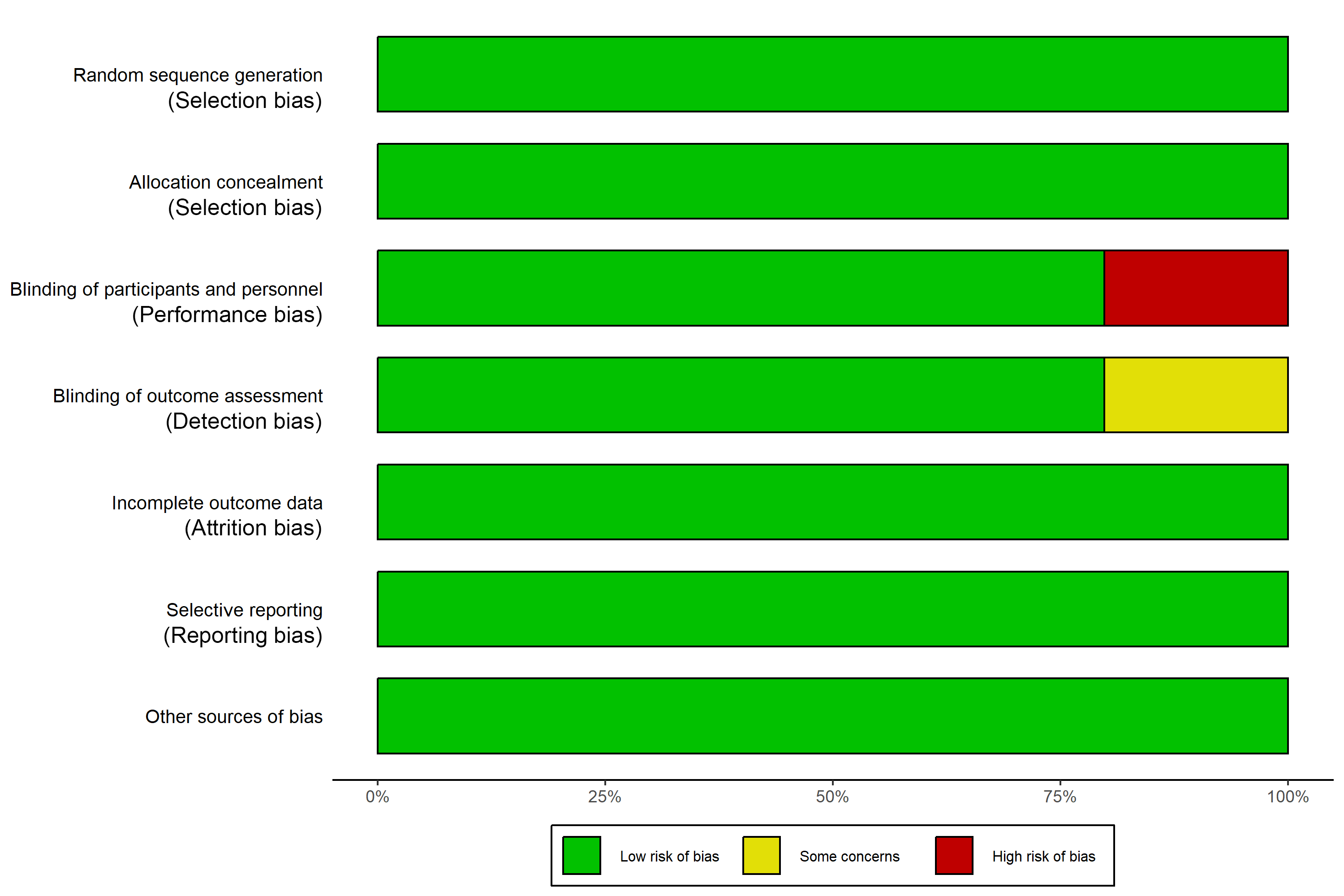
**

**Figure.S3.** Risk of bias summary.


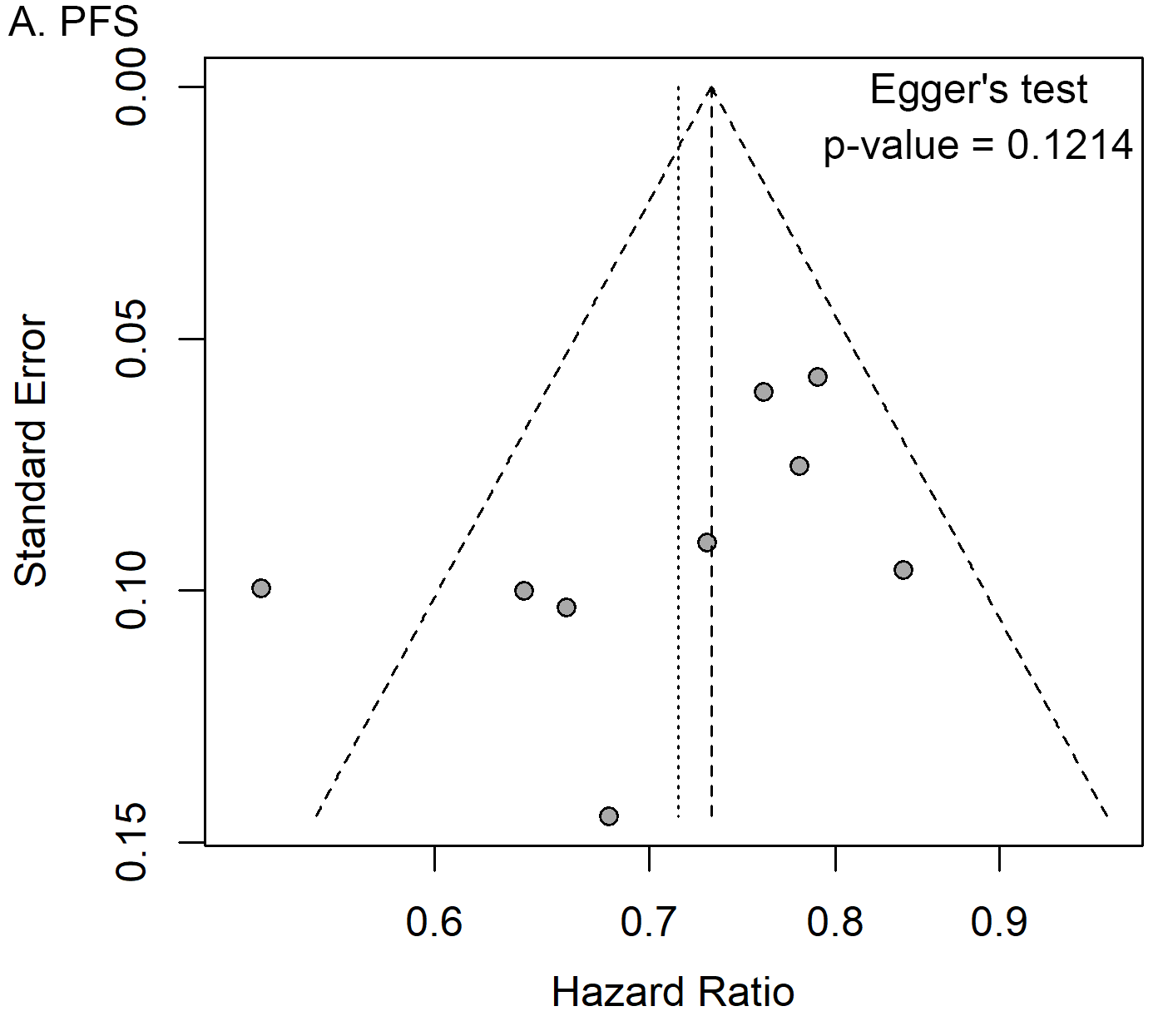

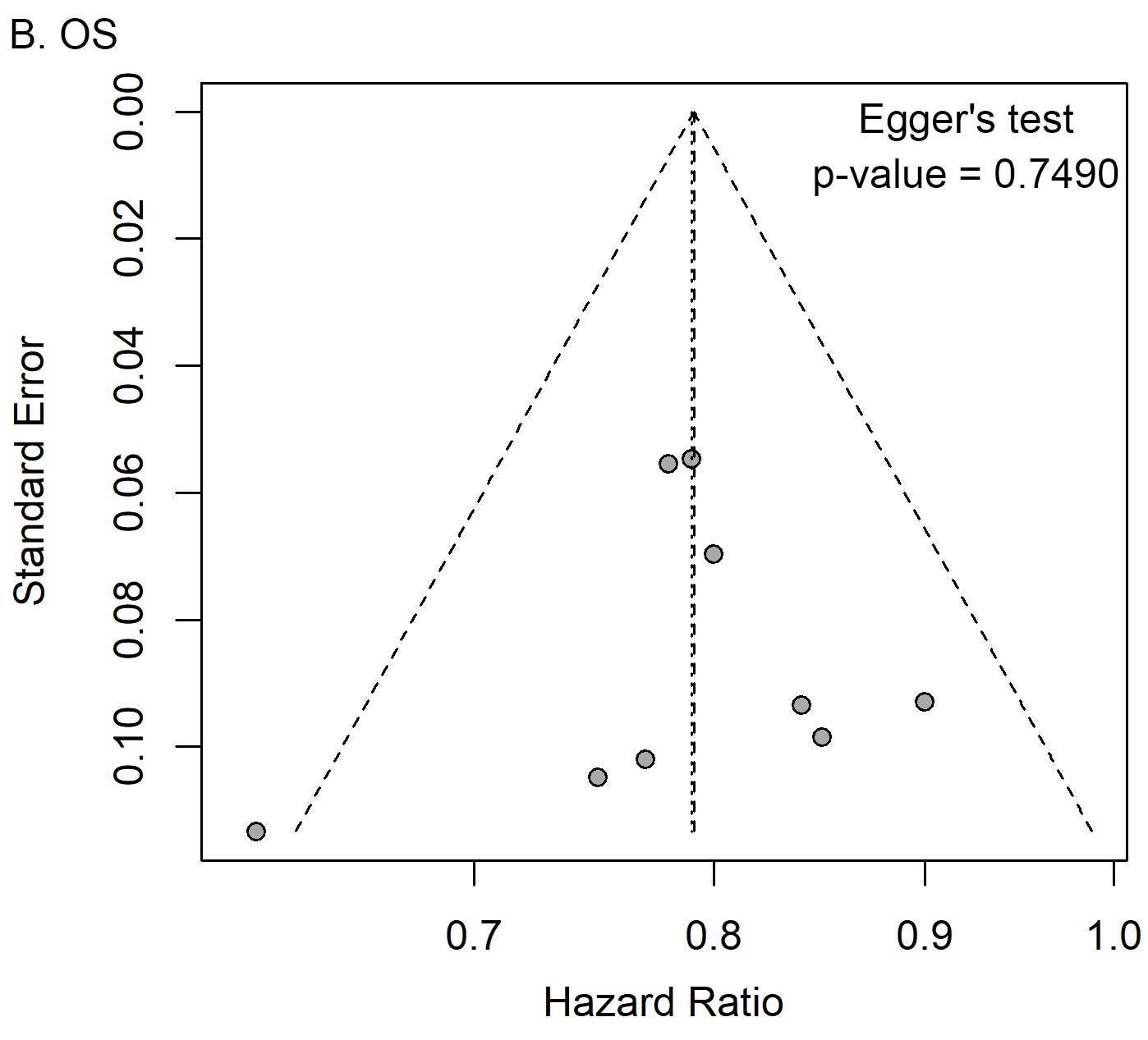


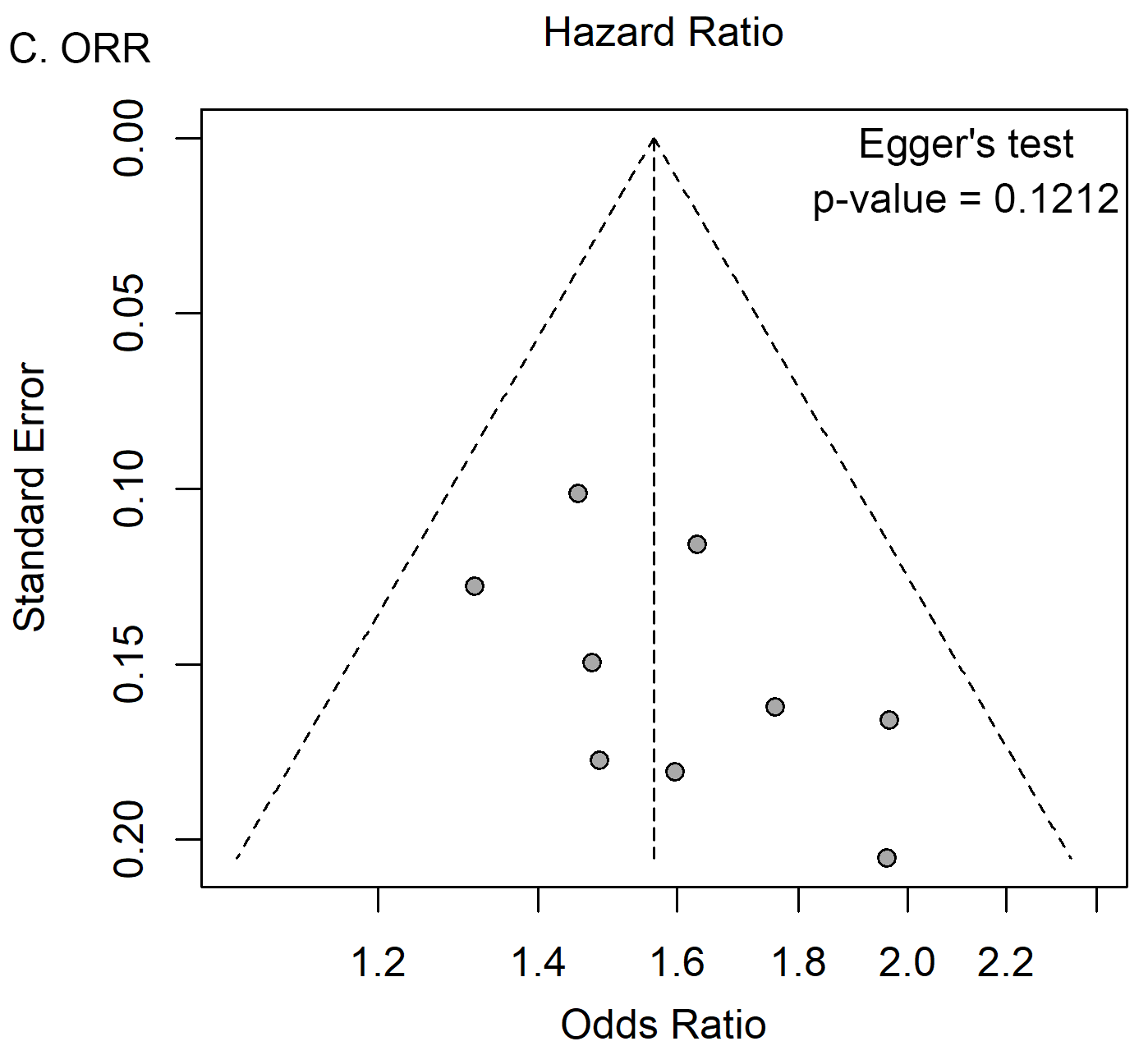


**Figure. S4.** Funnel plots and Egger’s tests for progression-free survival, overall survival, and objective response rate.


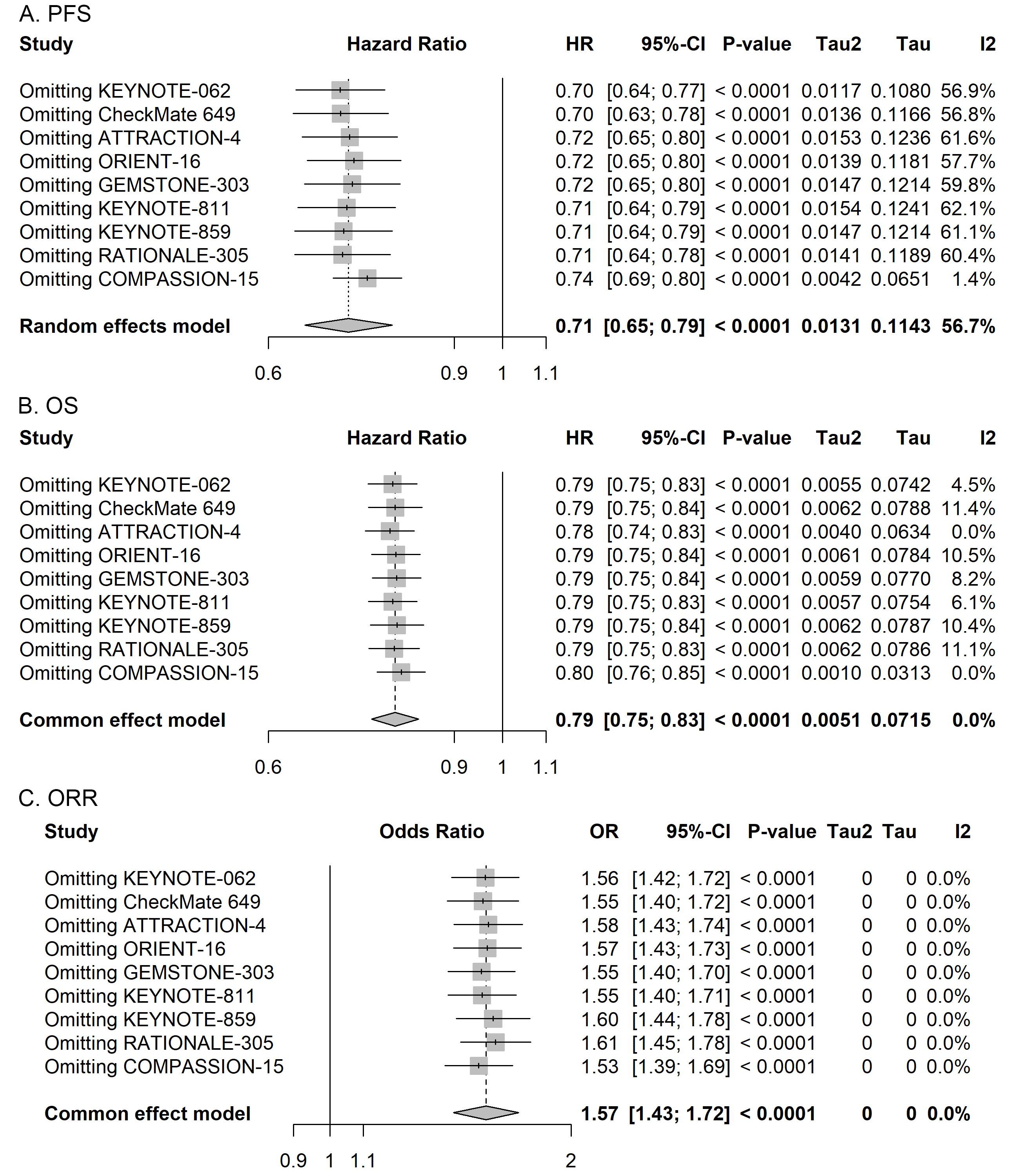


**Figure S5.** Sensitivity analyses for included studies on progression-free survival, overall survival, and objective response rate examined by leaving-one-out approach.
